# Analytical and Clinical Validation of a Remote Laboratory Test for Prothrombin Time and INR Determination in Patients with Antiphospholipid Antibody Syndrome and Systemic Lupus Erythematosus

**DOI:** 10.1021/acsomega.6c02189

**Published:** 2026-04-30

**Authors:** Gabriela Victória de Mello Jantzch, Rodrigo Ritzel Bernasconi, Milleny Germann Souza, Duane da Silva Moraes, Bruna Ferri Serafini, Leonardo Peterson dos Santos, Julia Konzen Moreira, Ricardo Machado Xavier

**Affiliations:** † Autoimmune Diseases Laboratory (LabDAI), 28124 Rheumatology Service, Hospital de Clínicas de Porto Alegre (HCPA), Porto Alegre 90035-003, Brazil; ‡ Postgraduate Program in Medicine: Medical Sciences, Federal University of Rio Grande do Sul (UFRGS), Porto Alegre 93022-715, Brazil; § Biosens Development and Biosensors Industry LTDA (BIOSENS), Sao Leopoldo 93022-750, Brazil; ∥ Polytechnic School, Systems Analysis and Development, University of Vale do Rio dos Sinos (UNISINOS), São Leopoldo 91501-970, Brazil; ⊥ Postgraduate Program in Chemistry, Federal University of Rio Grande do Sul (UFRGS), Porto Alegre 91501-970, Brazil; # Postgraduate Program in Mining, Metallurgical and Materials Engineering, Federal University of Rio Grande do Sul (UFRGS), Porto Alegre 93022-750, Brazil; ∇ School of Collective Health, University of Vale do Rio dos Sinos (UNISINOS), São Leopoldo 93022-000, Brazil

## Abstract

Antiphospholipid syndrome (APS) is an autoimmune disorder associated with antiphospholipid antibodies, leading to coagulation imbalance, thrombosis, and obstetric complications. It may occur as a primary condition or in association with systemic lupus erythematosus (SLE). Patients with APS often require long-term anticoagulation therapy, monitored by prothrombin time (PT) expressed as international normalized ratio (INR). Remote laboratory testing (RLT) devices may improve monitoring by providing faster and more convenient results; however, their accuracy in APS patients remains uncertain. This study aimed to validate a rapid and portable device (bSens.INR) for PT/INR measurement in patients with APS and SLE receiving warfarin, compared to a control group without these conditions. Accuracy, repeatability, and agreement with the standard laboratory method were evaluated according to established guidelines. A total of 40 PT/INR measurements were obtained using the bSens.INR and 20 using the standard method. Overall concordance was 77.5%, with lower agreement in APS/SLE patients (60%) compared to controls (95%). Approved results showed 100% repeatability. A strong positive correlation was observed between methods (Spearman *r* = 0.80, *p* < 0.0001), while Bland–Altman analysis indicated a bias of 0.26 INR units. The presence of antiphospholipid antibodies appears to interfere with PT/INR measurements obtained by the bSens.INR, reducing accuracy in APS and SLE patients. Although the device shows promising performance, its use in autoimmune populations should be approached with caution, reinforcing previous findings on the limitations of RLT in these conditions.

## Graphical abstract

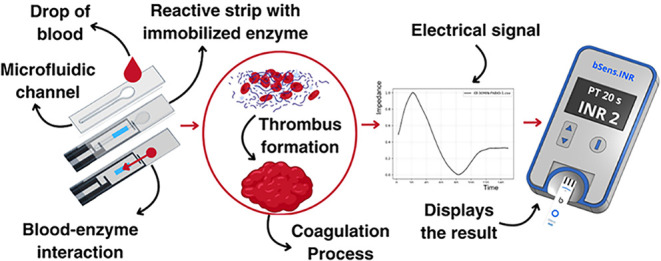

## 1 Introduction

Antiphospholipid syndrome (APS) is a systemic autoimmune disorder characterized by recurrent venous, arterial, or microangiopathic thrombosis and gestational morbidity in the presence of antiphospholipid antibodies (aPL). The primary target is β2-glycoprotein I (β2GPI), a plasma protein that binds to phospholipid surfaces and acts as a key anticoagulant cofactor. Around the 1990s, it was discovered that aPL primarily binds to β2GPI plasma protein when it is attached to phospholipids, rather than binding directly to the phospholipids themselves. The anti-β2GPI antibody test, using the Enzyme Linked Immunosorbent Assay (ELISA), now provides an additional method for detecting the presence of aPL. The binding of aPL to β2GPI on cell surfaces activates endothelial cells, monocytes, and platelets, leading to pro-inflammatory and pro-thrombotic phenotypes, complement activation, and ultimately thrombosis.^1^

The prevalence of the syndrome in the population ranges from 40 to 50 cases per 100,000 individuals. It is usually diagnosed in relatively younger individuals (30–40 years), with a higher prevalence among female patients; in a study of 1000 patients, 12.7% were diagnosed after the age of 50. When the disease affects males, it tends to have a later onset, with incidence occurring between 50 and 60 years of age.^2^

APS may occur as a primary condition or in association with other autoimmune diseases, particularly systemic lupus erythematosus (SLE). Approximately 30–50% of patients with APS present concomitant SLE or another associated autoimmune disease, with SLE being the most frequent. Although antiphospholipid antibodies are detected in approximately 30–40% of patients with SLE, only about 10–15% progress to clinically overt APS, characterized by thrombotic events and/or obstetric morbidity. The remaining 50–70% of APS cases are classified as primary APS, occurring in the absence of an underlying autoimmune disease.^2^

SLE is a chronic autoimmune inflammatory disorder characterized by heterogeneous clinical manifestations that may affect multiple organ systems, including the vascular system, central nervous system, lungs, skin, kidneys, and joints. This broad spectrum of involvement results from complex immunological and biological dysregulation. In addition to organ damage directly related to disease activity, patients with SLE are at increased risk of important comorbidities, such as hematologic malignancies, cardiovascular complications including accelerated atherosclerosis, skeletal conditions like osteonecrosis, and neuropsychiatric impairments, including cognitive dysfunction.^3^

SLE is diagnosed according to the 2019 American College of Rheumatology/European League Against Rheumatism (ACR/EULAR) classification criteria,^4^ which require the presence of antinuclear antibodies (ANA) at a titer of ≥1:80 as an obligatory entry criterion. Once this criterion is met, the diagnosis is established based on a weighted scoring system that incorporates clinical and immunological manifestations, with a total score of at least 10 points required for classification.

In contrast, APS is diagnosed based on the updated 2023 ACR/EULAR classification criteria,^5^ which integrate both clinical and laboratory findings. Clinical criteria include documented episodes of vascular thrombosis and/or obstetric complications, such as recurrent pregnancy loss or premature birth. Laboratory criteria require the persistent presence of antiphospholipid antibodies (anticardiolipin (aCL), anti-β2GPI, or lupus anticoagulant (LA)) detected on at least two occasions separated by a minimum interval of 12 weeks.

Treatment approaches may vary depending on the severity of the disease. However, the standard treatment consists of the use of vitamin K antagonists (VKAs), with regular laboratory monitoring required to maintain anticoagulation within recommended levels.^6,7^ The prothrombin time (PT), expressed as international normalized ratio (INR), is a key parameter in this monitoring process, used to measure the activation time of the coagulation cascade.^8^ This test, performed in clinical laboratories, assesses the effectiveness of VKA therapy and ensures that INR values remain within the therapeutic target, avoiding both the risk of thrombosis when values fall below the therapeutic range and the risk of bleeding when values exceed it. Moreover, VKAs are highly susceptible to influences from diet, lifestyle, and drug interactions, which further underscores the need for continuous laboratory monitoring.^9^

Current monitoring is centralized and relies on clinical laboratory infrastructure, which involves a multistep process: ordering the test, venous blood collection, sample referral to the laboratory, test execution, and report generation. This workflow introduces delays in decision-making, which is critical in time-sensitive conditions like coagulation disorders. The longer the interval between clinical data collection and therapeutic decisions, the greater the risk of the patient developing severe complications.^10^

An alternative to this scenario is a reliable remote laboratory testing (RLT), which aims to provide rapid results, facilitating decision-making. These tests have several applications, including in hemostasis.^11,12^ When used to measure PT, expressed as INR, the RLTs simplify procedures such as venous puncture and improve patient convenience. However, despite these advantages, their reliability remains uncertain, particularly in patients with APS. The presence of aPL in samples may interfere with the coagulation cascade, delaying thrombin formation and leading to inaccurate results, such as false positives or negatives.^13,14^ Although some devices have shown acceptable accuracy in patients without associated conditions, studies have reported significant discrepancies in INR results for APS patients.^15–17^ A study including 59 patients with antiphospholipid syndrome (APS) and 49 without APS demonstrated good diagnostic accuracy of RLT within low and high therapeutic INR ranges; however, adequate accuracy was not achieved in the supratherapeutic range, and the use of a standard laboratory method was recommended in such cases.^18^

Another study evaluated the CoaguChek RLT in comparison with standard laboratory testing in 13 APS patients and 28 non-APS patients. In the APS group, 54% of INR values deviated from the reference value, whereas in the non-APS group, deviations occurred in 32% of cases, demonstrating reduced accuracy in APS patients. The study also reported that several APS patients required repeated testing, as some had few or no comparable paired INR measurements. The authors recommended that RLT use in APS patients should only occur after individual comparison with standard laboratory testing before being adopted for routine monitoring.^15^

Additionally, another study does not recommend routine RLT use in APS patients, as a substantial proportion exhibited clinically unacceptable INR discrepancies, particularly in the INR > 4 range, which showed lower correlation with the standard method (*r* = 0.64) compared with other therapeutic ranges.^9^

This loss of sensitivity occurs primarily in remote laboratory tests (RLTs) because these devices typically rely on reduced sample volumes and simplified detection methods, making them more susceptible to interference from antiphospholipid antibodies (aPL), which delay thrombin formation and can affect the accurate measurement of prothrombin time.^15–17^

In parallel, recent advances in machine learning (ML) and artificial intelligence (AI) have demonstrated significant potential in enhancing the performance of biosensors, diagnostic platforms, and point-of-care technologies. These approaches have been successfully applied to improve signal processing, pattern recognition, and analytical accuracy in complex biological systems, including electrochemical and optical sensing platforms. Studies have shown that ML-assisted models can enhance sensitivity, reduce noise, and compensate for biological interferences, enabling more reliable interpretation of biochemical data. Furthermore, AI-driven strategies have been explored for optimizing material design, improving sensor interfaces, and enabling real-time data analysis in diagnostic applications. Such approaches are particularly promising in contexts where conventional analytical methods face limitations due to biological variability, as observed in APS patients.^19–24^

Therefore, the objective of this study is to validate a new RLT for determining PT and INR in patients with APS and SLE who are on oral anticoagulants (warfarin). For this purpose, the accuracy and precision of the results obtained with the Brazilian device under development, bSens.INR, were compared with those from patients receiving warfarin without APS or SLE, in order to assess whether there is a loss of sensitivity in the APS/SLE group and to identify the factors contributing to these differences. Previous studies have not applied uniform or comprehensive statistical methods to evaluate agreement between PT and INR detection methods. Therefore, the present study also aims to apply appropriate and robust statistical tests for analytical validation to ensure a rigorous assessment of method agreement and performance.

## 2 Materials and Methods

### 2.1 Study Design

This is a transversal diagnostic accuracy study conducted at the Hospital de Clínicas de Porto Alegre (HCPA), Brazil The institutional review board of the Universidade Federal do Rio Grande do Sul (UFRGS), HCPA, Brazil (registered under numbers 20230156) approved this study. The declaration of Helsinki principles was followed and all subjects gave written informed consent. We conducted this study in accordance with the 2015 STARD Reporting Guideline for Studies of Diagnostic Accuracy.^25^

All methodological procedures are summarized in the flowchart presented in Figure 1, depicting the study design, group allocation, sample size at each phase, laboratory assessments performed, and statistical analyses conducted. A comprehensive description of each methodological component is provided in the subsequent sections.

**Figure 1:**
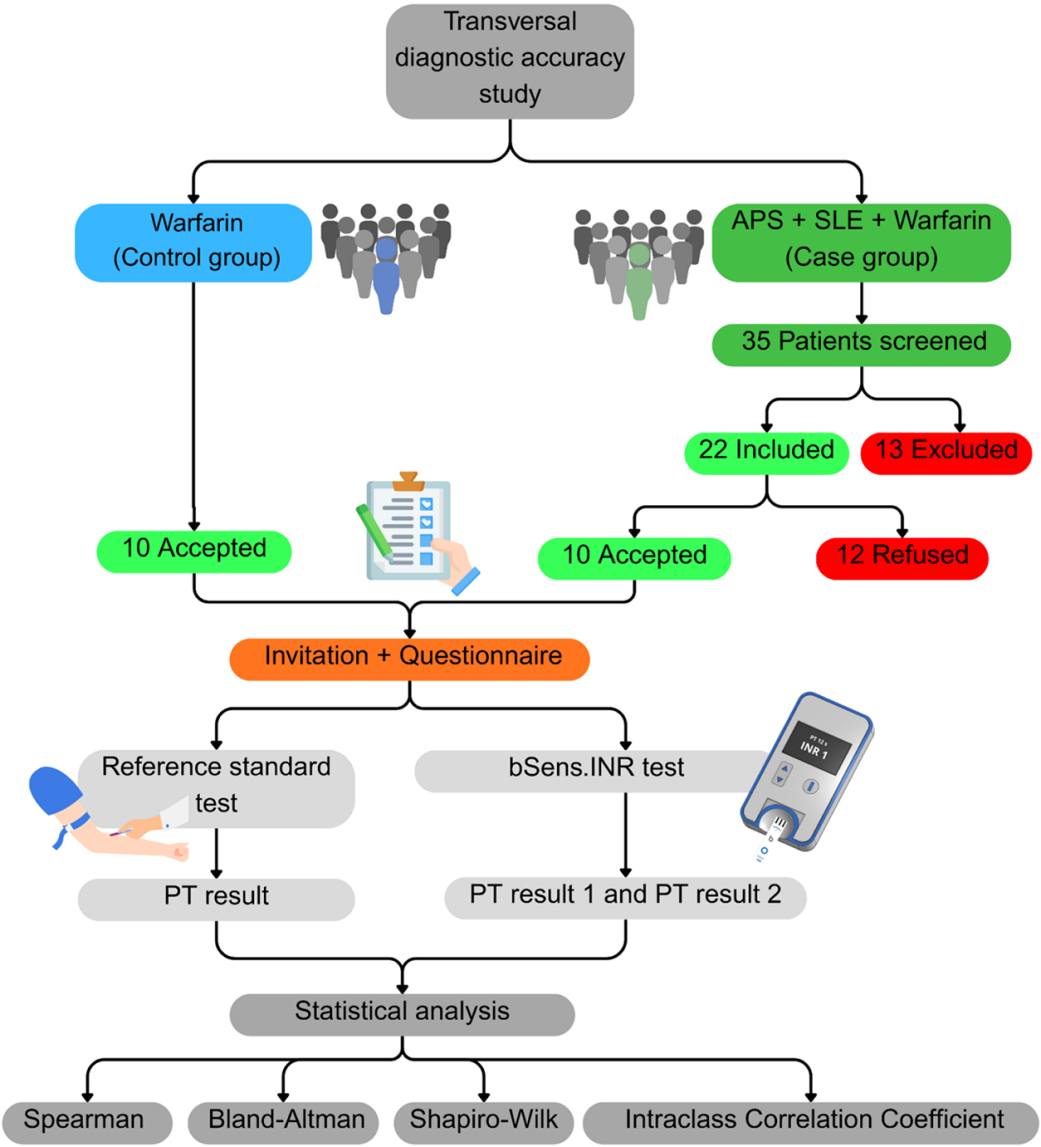
Flowchart of the analytical and clinical validation process of the remote PT/INR test in patients with antiphospholipid antibody syndrome and systemic lupus erythematosus.

### 2.2 Participants

Patients in the case group and control group were conveniently recruited between January and September 2024. The case group included patients diagnosed with APS and SLE, who were receiving oral anticoagulants (warfarin), who were recruited from the lupus clinic of HCPA. The control group consisted of patients on warfarin without a diagnosis of APS and SLE. Exclusion criteria comprised patients under 18 years of age or those who were not receiving oral anticoagulants, as the study focused on evaluating the performance of an RLT in patients undergoing anticoagulant therapy.

Based on these criteria, total of 71 patients with APS associated with SLE were identified. Of these, 36 were not receiving warfarin therapy, 1 patient was under 18 years of age, and 12 did not authorize participation in research studies. Consequently, 22 patients met the inclusion. Among them, 12 declined to participate, and 10 agreed to enroll in the study, completing a questionnaire followed by biological sample collection.

The relatively small sample size in the case group reflects not only the low prevalence and clinical heterogeneity of APS associated with SLE, but also the inherent challenges in recruiting patients under controlled clinical conditions and strict eligibility criteria. While this limited number of cases may impact the generalizability of the findings, the study was primarily designed to assess analytical performance and method agreement, for which the applied statistical approaches remain appropriate and robust even in smaller cohorts. To ensure comparability between groups and minimize selection bias, an equal sample size was predefined for the control group, adopting a 1:1 allocation ratio, that is, 10 volunteers in the case group (patients using warfarin with APS and SLE) and 10 in the control group (patients using warfarin without APS and SLE).

### 2.3 Compared Analytical Methods

Initially, two capillary blood samples were collected from each participant for analysis using the bSens.INR device. Subsequently, a venous blood sample was collected and stored in a tube containing sodium citrate (0.109 and 0.105 mol/L [3.2%]), following Clinical and Laboratory Standards Institute (CLSI) guidelines, with a blood-to-citrate ratio of 9:1 for conventional laboratory testing. Clinical characteristics such as age (years), disease duration (years), and self-reported race/color (white, black, brown) were assessed through medical record review. Duration of warfarin use (years), weekly warfarin dose (mg), frequency of PT testing (days), comorbidities, thrombotic events (yes/no), hemorrhagic events (yes/no), and the presence of antiphospholipid antibodies were recorded by the research team during the consultation. Clinical data were used to characterize the demographic profile of the study participants and to support the analyses and discussion of the results.

#### 2.3.1 Testing with the bSens.INR Device

Testing with the bSens.INR device, a Brazilian-developed technology by Biosens currently under regulatory review by Brazilian Health Regulatory Agency ANVISA and subject to patent application, was carried out under the supervision of a qualified healthcare professional. During the collection procedure, a test strip containing immobilized thromboplastin reagent was inserted into the device, initiating the heating process. Once the strip reached the ideal temperature for the reaction (33.5 °C), the device signaled for the deposition of a capillary blood drop. After the biological sample filled the microfluidic channel, ensuring contact between the blood, reagent, and sensors, the coagulation reaction began. The sample’s viscosity was measured over a 3 min period using an electrochemical impedance technique, and the result, expressed in seconds and INR, was displayed on the device’s screen. The procedure is illustrated in Figure 2.

**Figure 2:**
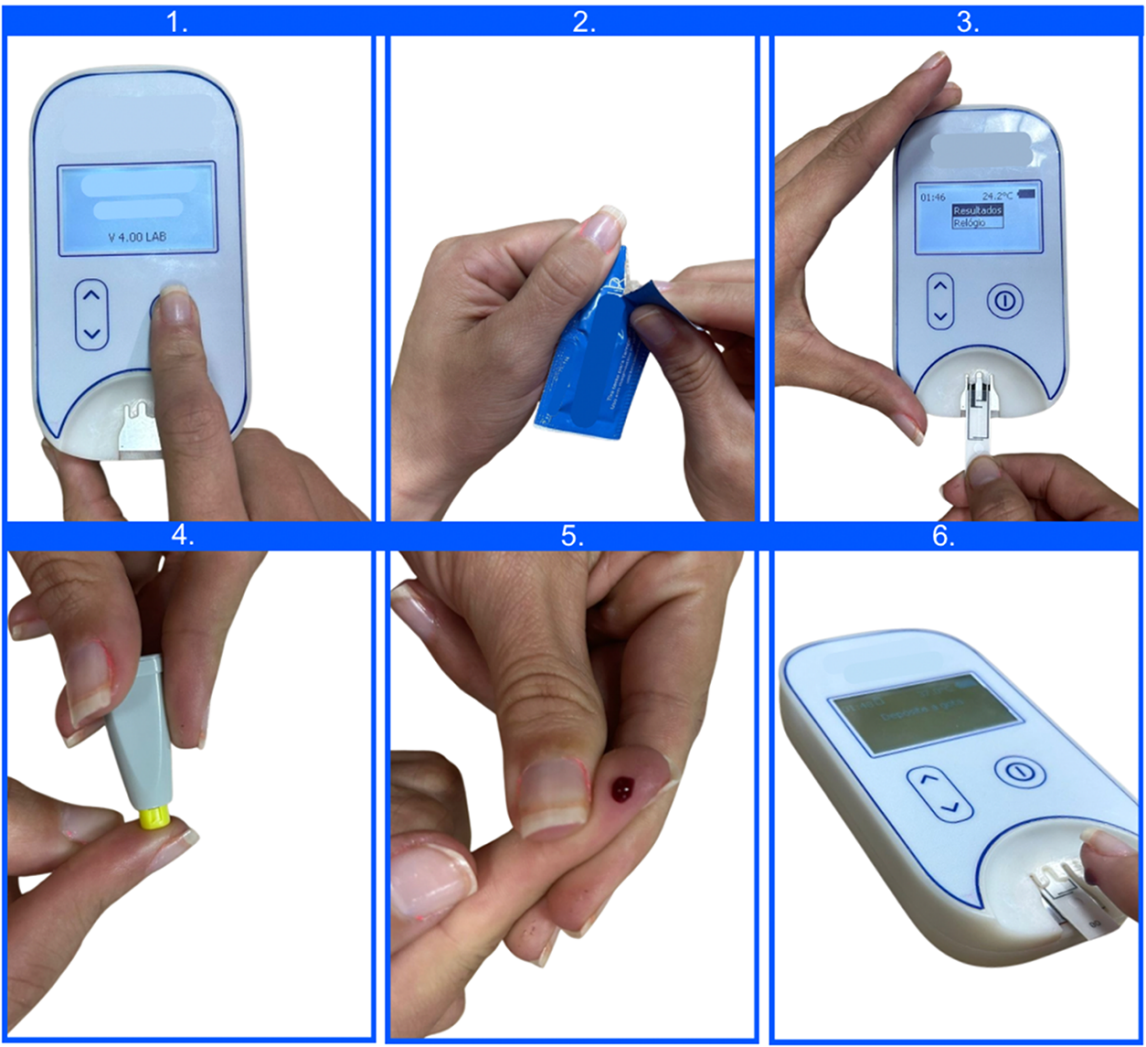
Operating principle of the bSens.INR device. 1. Device activation. 2. Removal of the test strip from the packaging. 3. Insertion of the test strip into the device. 4. Capillary puncture using a lancet. 5. Fingertip blood collection. 6. Addition of a drop of blood onto the test strip already inserted into the device to initiate the test. Photograph courtesy of Biosens. Copyright 2025.

This technique is based on the principle that the electrical impedance of a sample changes as its physical and chemical properties are altered. In the case of blood coagulation, as the sample changes from a liquid state to the formation of a clot, the movement of ions and charged particles becomes increasingly restricted. The device applies a small alternating current across the electrodes, and the resulting voltage response is measured. Changes in impedance over time directly reflect the increasing resistance to ionic flow caused by clot formation, allowing the system to monitor the kinetics of coagulation. By correlating these impedance changes with standard coagulation times, the device can calculate the PT in a rapid and quantitative manner, using standardized mathematical functions, and INR is subsequently calculated considering the International Sensitivity Index (ISI) and a normal PT range of 10–14 s.

#### 2.3.2 Reference Standard Test

The venous blood sample, used for the conventional laboratory test and analyzed in a clinical laboratory accredited by the National Quality Control Program (NQCP), was evaluated using an optical coagulometry in the ACL TOP 750 equipment. For PT measurement, the venous sample was centrifuged to separate the plasma from blood cells. In the coagulometer, the plasma sample was mixed with a reagent containing calcium and human thromboplastin, which reverses the action of the citrate anticoagulant in the storage tube, initiating the coagulation cascade. The coagulometer then monitors the reaction as the coagulation process progresses, detecting changes in viscosity until clot formation occurs.

This approach differs from the bSens.INR device, which detects changes in blood viscosity during clot formation by monitoring variations in electrical impedance, whereas the standard coagulation test determines clot formation by measuring the gradual increase in light absorption as the clot forms.

### 2.4 Statistical Analysis

The sample calculation was based on Taylor et al. (2017) using software developed by researchers at the University of São Paulo (USP), which provides a standard error of the limit of agreement of 95%, with a 95% confidence level, and considers a minimum acceptable difference of ±0.5 INR units (with the addition of 10% to account for potential losses and refusals). To assess the agreement between the standard optical mechanical coagulometric method and the electrochemical method of the bSens.INR device, a sample size of 15 individuals per group (*n* = 30) was suggested.^15,26–28^ The Shapiro-Wilk method was used to test for normality. Results are expressed as mean ± standard deviation (SD), median (interquartile range, IQR), and number (%), as appropriate.

To assess the diagnostic accuracy of the bSens.INR device in measuring PT and INR, predetermined acceptable deviation limits were applied, based on the INR values obtained from the conventional laboratory test. These limits were calculated according to the criteria established by International Organization for Standardization (ISO) 17593:2022. For INR < 2, the acceptable deviation was ±0.4 INR units, for 2 ≤ INR < 4.5, a deviation of ± 20% was considered; and for INR ≥ 4.5, a deviation of ±25% was acceptable. The INR result obtained from the bSens.INR was considered accurate if it fell within these predefined limits.^27^

Additionally, the repeatability of the results obtained from the bSens.INR device was assessed by analyzing the duplicate measurements for each participant. This evaluation followed the guidelines of ISO 17593:2022, which recommends determining the acceptable interval between replicates for the same patient, using the following equation: mean of the replicates × 0.10 × 4.2, where 0.10 represents a 10% coefficient of variation and 4.2 is a constant.^27^

A Spearman analysis was performed to identify the relationship between the INR result of the reference standard test compared to the bSens.INR test. The correlation strength was determined from the classification suggested by Dancey and Reidy: *r* = 1.0 indicates perfect association; 0.7 ≤ *r* ≤ 0.9, strong association; 0.4 ≤ *r* ≤ 0.6, moderate association; 0.1 ≤ *r* ≤ 0.3, weak association; and *r* = 0, no association.^28^

Bland-Altman analysis was conducted to graphically demonstrate the agreement between the methods, verifying whether the differences between measurements were within acceptable limits. Finally, an Intraclass Correlation Coefficient (ICC) analysis was performed to evaluate the reliability between the INR measured by the bSens.INR and the standard method. The significance level was set at *p* ≤ 0.05 for all analyzes. Statistical analyzes were performed in Statistical Package for Social Sciences (SPSS) 17.0.

## 3 Results and Discussion

The study included 20 participants: 10 patients with APS and SLE who were on oral anticoagulant therapy (warfarin), named as the case group, and 10 participants using oral warfarin without APS and SLE, named as the control group. Table 1 demonstrates the characterization of the population studied through the demographic data collected (sex, race and age) and clinical aspects in relation to each group evaluated. From the data, it is possible to observe that the case group has a female predominance due to the higher prevalence of SLE in women.^2^ As expected, this group presents a high prevalence of thrombotic events.

**Table 1:** Study Population Characteristics

	case group	control group
women (%)	100	60
men (%)	0	40
race - white (%)	90	90
race- mixed race (%)	10	10
age in years (mean ± SD)	42 ± 13	52 ± 13
hypertension (%)	60	80
time of use of Warfarin medication (years ± SD)	9 ± 5	9 ± 6
weekly Warfarin dose (mean in mg ± SD)	53 ± 25	44 ± 25
frequency of PT laboratory testing (days ± SD)	24 ± 10	37 ± 14
patients with thrombotic events (%)	60	10
patients with hemorrhagic events (%)	10	30

From the experimental test, it was possible to obtain 40 PT and INR results on the bSens.INR device due to duplicate evaluations, and 20 results from the conventional laboratory test. Through the medical records, it was also possible to identify the antibodies present in the patients in the case group. All data are presented in Table 2. The diagnostic accuracy analysis, which included both groups, demonstrated that 77.5% of the results obtained through the bSens.INR RLT were approved for accuracy according to the ISO criteria. When performing the same analysis by separating the groups, a reduction in accuracy was observed in the case group, which showed 60% accuracy compared to the control group, which showed 95% of results approved for accuracy. The results also demonstrate that the bSens.INR only showed accuracy deviations in tests performed on patients from the case group with high levels of aCL. Furthermore, in the repeatability analysis, which included only the results that met the accuracy criteria in both groups (31 out of 40 results), the precision rate was 100%.

**Table 2:** Data Obtained from RLT Tests and Standard Method Tests^a^

group-code	antibodies	PT 1 and 2 bSens.INR	INR 1 and 2	standard PT test	INR	limit – *	limit + *
case-1	aCL (high)	50.00	5.04**	31.30	2.78	2.22	3.33
		47.50	4.73**				
case-2	LA+;	22.70	1.95**	44.20	3.97	3.17	4.76
	aCL(high);	18.60	1.54**				
	anti-β2GPI (moderate)						
case-3	LA+;	17.10	1.39	13.20	1.14	0.74	1.54
	anti-β2GPI (moderate)	18.60	1.54				
case-4	LA+;	16.80	1.36**	27.50	2.43	1.94	2.91
	aCL (high)	20.80	1.76**				
case-5	aCL (moderate)	28.10	2.52	28.00	2.48	1.98	2.97
		25.30	2.22				
case-6	Anti-β2GPI (high)	20.73	1.75	15.80	1.37	0.97	1.77
		17.20	1.40				
case-7	aCL (high)	24.50	2.14**	40.00	3.56	2.84	4.27
		26.40	2.34**				
case-8	aCL (moderate)	20.30	1.71	18.90	1.65	1.25	2.05
		20.90	1.77				
case-9	aCL (moderate)	35.60	3.35	33.70	3.00	2.40	3.60
		29.00	2.62				
case-10	aCL (moderate)	22.10	1.89	18.40	1.65	1.25	2.05
		18.00	1.48				
control-11	NA	14.80	1.17	13.10	1.13	0.73	1.53
		15.40	1.23				
control-12	NA	34.80	3.26	37.60	3.36	2.68	4.03
		33.90	3.16				
control-13	NA	37.10	3.52	50.00	4.55	3.41	5.68
		43.70	4.28				
control-14	NA	24.20	2.11	27.10	2.44	1.95	2.92
		26.50	2.35				
control-15	NA	22.10	1.89	26.30	2.37	1.89	2.84
		24.20	2.11				
control-16	NA	23.20	2.00	23.30	2.02	1.61	2.42
		21.80	1.86				
control-17	NA	38.90	3.73	54.40	4.96	3.72	6.20
		35.80	3.37**				
control-18	NA	27.30	2.44	29.90	2.70	2.16	3.24
		29.10	2.63				
control-19	NA	27.70	2.48	29.00	2.66	2.12	3.19
		29.32	2.65				
control-20	NA	22.92	1.97	24.90	2.23	1.78	2.67
		22.31	1.91				

a *The INR acceptability limits were established based on the guidelines of the ISO 17593:2022 standard, using the values obtained by the standard laboratory method of INR measurement. PT 1 refers to the first replicate of the sample collected from the patient’s fingertip. PT 2 refers to the second replicate of the sample collected from the patient’s fingertip. Limit (−) corresponds to the minimum acceptable INR value, while the Limit (+) represents the maximum acceptable value within the criteria defined by the standard. ** Results outside the established limits, that is, results that do not agree with the standard method result. LA: + (present), – (absent) and aCL/anti-β2GPI: moderate (≥40-79U) and high (≥80U) according to criteria.^4,5^ NA: (not available).

The Spearman correlation analysis revealed a strong, positive, and statistically significant correlation between the INR result of the standard laboratory method compared to the bSens.INR (*r* = 0.80, *p* < 0.0001). As the INR of the bSens.INR increases, the results of the standard method tend to increase consistently. The Bland-Altman analysis revealed a bias of 0.26, indicating that the bSens.INR method tends to provide INR values that are 0.26 units higher or lower than the standard method, which may indicate that the bSens.INR could overestimate INR values compared to the standard laboratory test. Most data points fell within the limits of agreement (±2 standard deviations), suggesting overall concordance between the bSens.INR and the laboratory method as shown in Figure 3. However, four results from the case group (green) were outside the acceptable limits, which were also flagged in the accuracy analysis. No deviations were observed in the control group (blue).

**Figure 3:**
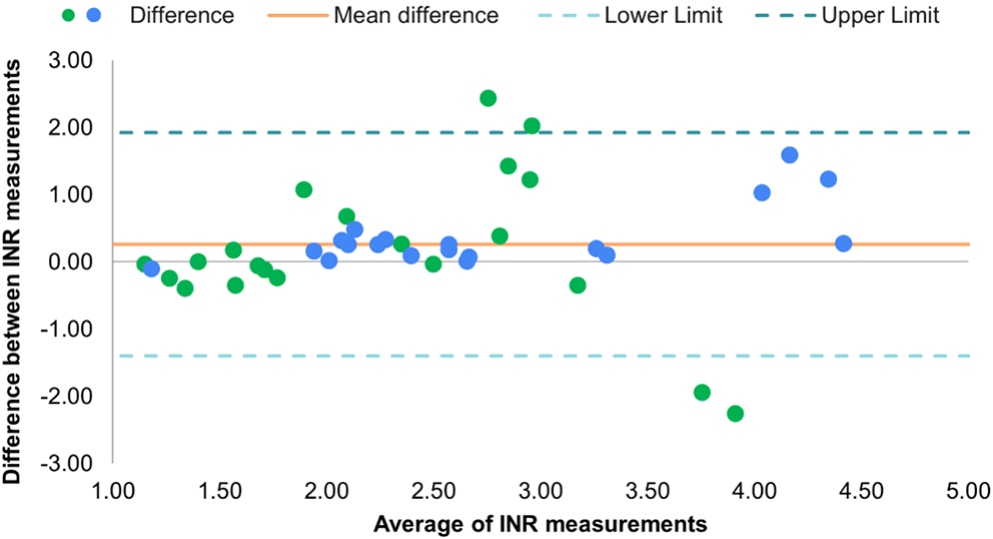
Bland-Altman analysis of the case and control groups. The points represent the difference between the INR measured by bSens.INR and the standard method for the case group (in green) and the control group (in blue). The solid orange line represents the mean difference between the evaluated methods. The dashed lines represent the limits of agreement of ± 2 standard deviations.

Regarding the ICC, the value obtained was 0.64 (95% Confidence Interval: 0.29 to 0.84) indicating moderate consistency between the INR measurements performed by the bSens.INR and the standard method.

Although the bSens.INR demonstrates good precision in most cases, the observed bias, low accuracy and low agreement in the case group suggest that the device may not be ideal for exclusive use in monitoring these patients. This finding has been corroborated by other studies that evaluated the precision and accuracy of RLT devices like CoaguChek in patients with APS, which found significant variations compared to conventional laboratory tests. These results are consistent with the observation that the bSens.INR exhibits low accuracy in the case group, as noted by Taylor et al. This reinforces that RLT devices, such as bSens.INR, CoaguChek, Pro Time and qLabs, are unable to detect PT and INR in patients with APS with the same accuracy as they detect these parameters in individuals without this combination of diseases.^9,15–18,29^

An analysis of the antibodies present in the case group, with respect to PT and INR results that deviated from the accuracy limits established by the standard method, revealed that increased levels of aCL antibodies were the most evident common factor, suggesting a possible interference in the results obtained by the bSens.INR device. Two other studies reported similar findings with remote laboratory testing (RLT) devices, although involving different antibodies. In a study with 33 participants with APS, anti-β2GPI antibody levels were associated with interference in INR results from these RLT devices, particularly for INR values >3. Patients with high antibody titers were more likely to have inconsistent results, suggesting that these antibodies may distort the readings.^17^

In the present study, evidence aligns with the literature: the patient with isolated anti-β2GPI exhibited an INR < 3 without significant deviation. Furthermore, a patient with both anti-β2GPI and LA+ showed no deviation from the expected result, indicating that the presence of LA+ may not interfere with the measurement, particularly for INR < 3. However, no patient presented isolated LA+, which limits the ability to draw definitive conclusions. Therefore, it can be concluded that the antibody with the greatest impact on method concordance was aCL at elevated levels.

Another study with 59 APS patients investigated antibody presence and found that anti-β2GPI, specifically, had a negative impact on INR results obtained by RLT. In some patients with high LA levels, coagulation time could not be measured with RLT, possibly due to interactions with thromboplastin on the test strips, where the binding of antibodies to the phospholipid in thromboplastin may alter the formation of the coagulation activation complex, resulting in erroneous INR readings, either overestimating or underestimating the true value. Another hypothesis of interference is that antiphospholipid antibodies may form immune aggregates with lipids or proteins in the blood, and these aggregates could affect the measurements, as aggregation may interfere with blood viscosity or with the detection of clot formation in the devices.^18^

One important limitation of this study is the relatively small sample size in the case group, which may limit the external validity and generalizability of the findings. This constraint is largely due to the low prevalence of antiphospholipid syndrome associated with systemic lupus erythematosus, in addition to the strict inclusion criteria, particularly the requirement for patients to be under continuous warfarin therapy.

Despite these limitations, this study provides a preliminary evaluation of the performance of the bSens.INR device in a clinically relevant population. Nevertheless, further investigations with larger and more heterogeneous cohorts are necessary to validate these results and support broader clinical applicability.

The findings of this study provide additional evidence that RLT devices for PT and INR determination have important limitations when applied to patients with antiphospholipid antibody syndrome. The reduced accuracy and agreement observed in this population reinforce the need for cautious interpretation of results and highlight the importance of method-specific validation before routine clinical use in APS patients.

## 4 Conclusion

In conclusion, the findings of this study demonstrate that the bSens.INR device exhibits significantly reduced diagnostic accuracy for PT and INR measurement in patients diagnosed with Systemic Lupus Erythematosus and Antiphospholipid Syndrome compared to control groups. Distinguishing itself from previous research, which often lacked uniform or comprehensive statistical frameworks, this study employed a robust suite of analytical validation tests to ensure a rigorous assessment of method agreement. By utilizing these stringent statistical standards, our results provide a more definitive characterization of the interference likely mediated by antiphospholipid antibodies on portable electrochemical sensors. Consequently, while RLT methodology offers significant logistical advantages, their clinical application in SLE and APS populations must be approached with caution. These findings underscore the urgent need for further research to refine sensor sensitivity, ensuring that portable diagnostic tools meet the high precision standards required for the safe management of high-risk autoimmune patients.

Furthermore, despite the limitation of a relatively small sample size due to the specific local availability of patients, this study provides a robust statistical foundation that can serve as a benchmark for future validation research. Our methodological approach reinforces findings from previous studies comparing laboratory-based PT standards with RLT, while offering a more rigorous framework for evaluating analytical agreement. By bridging these data, the present study contributes to a more reliable understanding of the technological constraints inherent in portable coagulometers when applied to complex clinical profiles.

Future research should focus on expanding the sample size, particularly within APS and SLE populations, to enable stratified analyses based on antiphospholipid antibody profiles, including lupus anticoagulant and anti-β2GPI titers. Longitudinal studies are warranted to assess intraindividual variability and to determine the clinical impact of INR discrepancies on therapeutic decision-making and patient outcomes. In addition, mechanistic investigations exploring the interaction between antiphospholipid antibodies and electrochemical detection systems may help elucidate the sources of analytical interference. From a technological perspective, the incorporation of advanced data processing approaches, such as machine learning and artificial intelligence algorithms, represents a promising strategy to mitigate biological interferences and improve measurement accuracy.

Furthermore, the integration of remote laboratory testing devices with artificial intelligence–driven analytics and machine learning models may enable more personalized therapeutic monitoring, allowing dynamic adjustment of anticoagulation management based on individual patient profiles and longitudinal data. Such approaches have the potential to enhance patient engagement and adherence to regular INR monitoring, ultimately improving the proportion of time within the therapeutic range. Consequently, these advancements may contribute to maintaining patients within the target INR range, thereby reducing the risk of adverse events, including thrombotic and hemorrhagic complications.

## Data Availability

The data sets generated and/or analyzed during the current study are available in the repository https://docs.google.com/spreadsheets/d/14flnctrg_kqcGvEG3HFKEF2dfZMmU9_U/edit?usp=sharing&ouid=107025160705232668239&rtpof=true&sd=true.

## Abbreviations

ACR: American College of Rheumatology
ANA: antinuclear antibodies
ANVISA: Agência Nacional de Vigilância Sanitária
APS: antiphospholipid syndrome
aCL: anticardiolipin
aPL: antiphospholipid antibodies
ML: machine learning
AI: artificial intelligence
β2GPI: β2-glycoprotein I
CLSI: Clinical and Laboratory Standards Institute
ISI: international sensitivity index
ELISA: enzyme Linked Immunosorbent Assay
EULAR: European League Against Rheumatism
HCPA: Hospital de Clínicas de Porto Alegre
ICC: intraclass correlation coefficient
INR: international normalized ratio
ISO: International Organization for Standardization
LA: lupus anticoagulant
NQCP: National Quality Control Program
PT: prothrombin time
RLT: remote laboratory testing
SD: standard deviation
SLE: systemic lupus erythematosus
SPSS: statistical package for social sciences
UFRGS: Universidade Federal do Rio Grande do Sul
USP: University of São Paulo
VKAs: vitamin K antagonists
